# Utilization of non-pneumatic anti-shock garment and associated factors for postpartum hemorrhage management among health care professionals’ in public hospitals of Jimma zone, south-West Ethiopia, 2019

**DOI:** 10.1186/s12978-020-0891-6

**Published:** 2020-03-17

**Authors:** Gadisa Bekele, Gemechu Terefe, Makeda Sinaga, Sena Belina

**Affiliations:** School of Nursing and Midwifery, Faculty of health Sciences, Institute of health, Jimma University, P.O.BOX:378 Jimma, Ethiopia

**Keywords:** Postpartum hemorrhage, Anti-shock garment, Maternal mortality

## Abstract

**Background:**

Non- pneumatic anti-shock garment is a unique, life -saving first -aid device made of neoprene and velcro, which is used for treatment of women with postpartum hemorrhage. Maternal mortality in the world still very high and postpartum hemorrhage is the leading cause of maternal mortality worldwide.

**Objective:**

This study was aimed to assess the utilization of non-pneumatic anti-shock garment and associated factors for postpartum hemorrhage management.

**Methods:**

Facility based cross-sectional studies design both quantitative and qualitative data collection methods were employed among 210 health care professionals and 10 key informants respectively. Bivariable and multivariable logistic regression was done to identify factors associated with non-pneumatic anti-shock garment utilization. Qualitative data was transcribed, translated and triangulated with quantitative findings.

**Results:**

Seventy six (36.2%) of the respondents used non-pneumatic anti-shock garment in their hospitals for management of post-partum hemorrhage. Having good knowledge [(AOR = 3.96, 95% CI: (1.67, 9.407)], having positive attitude [(AOR = 3.54, 95% CI: (1.37, 9.13)], attending training [AOR = 13.156, 95% CI: (4.81, 36.00], having two and above non-pneumatic anti-shock garment at their hospitals [AOR = 8.7, 95% CI: (2.89, 26.20)] were significantly associated with utilization of non-pneumatic anti-shock garment**.** … “I didn’t use non-pneumatic anti-shock garment for the management of postpartum hemorrhage complication before because I have no training and experience how to use it.”

**Conclusion:**

The utilization of non-pneumatic anti-shock garment for the management of postpartum hemorrhage was low. Having positive attitude, having good knowledge and training on non-pneumatic anti-shock garment were statically associated with its use. The health care professionals that involved in the maternity service should be trained on how to use this important garment in the management of postpartum hemorrhage.

## Plain English summary

The leading cause of maternal mortality (deaths from pregnancy and childbirth related complications) is obstetric hemorrhage in which a woman bleeds heavily, most often immediately after giving birth. A woman somewhere in the world dies every 4 min from this kind of complication**.**

The Non-pneumatic Anti-shock Garment is a first-aid device used to stabilize women who are suffering from obstetric hemorrhage and shock. It is a simple device helps women survive delays in getting to a hospital and getting the treatment that they need. It can be applied by anyone after a short, simple training.

Respondents were asked on utilization of Non-pneumatic Anti-shock Garment for Postpartum Hemorrhage Management. Knowledge and attitude of care providers was assessed quantitatively and additional information was collected from key informants qualitatively to explore factors in detail.

Of 210 Seventy six (36.2%) used NASG for postpartum hemorrhage management while 63.8% never applied it for postpartum hemorrhage complication management. The reasons stated by respondents those did not use non-pneumatic anti-shock garment, (35.4%) were due to lack of experience and (23.8%) due to lack of awareness about availability of the instruments.

Periodic training for health professionals on the use of non-pneumonic anti-shock garment for management of obstetric hemorrhage is important.

## Introduction

Non- pneumatic Anti-Shock Garment (NASG) is a unique, life -saving first -aid device made of neoprene and velcro, which is used for women with obstetric hemorrhage. It can be applied by anyone, even those without medical training. Non-pneumatic anti-shock garment has a unique role in hemorrhage and shock management because it is meant to be used with, other technologies. Currently, it is the only tool first aids in stabilizing pulse and blood pressure after a woman has gone into shock from obstetric hemorrhage. Non-pneumatic anti-shock garment can reverse shock and can be used at the same time with other hemorrhage and shock treatments, such as: uterine massage, uterotonics, blood transfusions, vaginal procedures and surgery, uterine balloon tamponed [1, 2]. Non-pneumatic anti-shock garment was developed in 1970s by the United States (US) National Aeronautics and Space Administration (NASA)/ Ames [3].

International Confederation of Midwives (ICM) and the International Federation of Gynecology and Obstetrics (FIGO) recommended the non-pneumatic anti-shock garments as it reduce mortality [4].

The non-pneumatic anti-shock garments like bottom half suit and consists of three pairs the first of segments, the first segments are placed around the patient’s legs and the second three segments are placed around her pelvis and abdomen during the application for postpartum hemorrhage. A ball in segment 5 is placed over the woman’s umbilicus (belly button, navel), adding more pressure. Non-pneumatic anti-shock garment is to be worn until the patient’s vital sign have stabilized for 2 h or more, include blood loss < 50 ml/hr., pulse < 100 bpm and systolic blood pressure > 100 mmhg. Removing of NASG begins from lowest segments and proceeds upward by allowing 15 min between removals each segment for redistribution of blood. But if her hemodynamic status has not become unstable use the “Rule of **20**”, that means her pulse has not increased by more than 20 bpm and her systolic blood pressure has not decreased by more than 20 mmHg. If pulse and blood pressure remain stable, open the next segment pair [5, 6].

Non-pneumatic anti-shock garment introduced in Ethiopia by Clinton Health Access Initiative (CHAI) as a pilot project to prevent postpartum hemorrhage in Oromia and Tigray regions [7].

Using non-pneumatic anti-shock garment can reduce the time to recovery from shock, decrease blood loss to external, increases both external and internal abdominal pressure [8].

“Maternal mortality is the death of a woman during pregnant or within 42 days of termination of pregnancy, irrespective of the duration and site of the pregnancy, from any cause related to or provoked by the pregnancy but not from accidental or incidental causes” [9]. The death of a woman in childbirth or from pregnancy-related events is a devastating experience for the woman’s family, her partner and her surviving children and is also devastating for all involved in her care [10].

The maternal mortality ratio in developing countries in 2015 is 239 per 100,000 live births versus 12 per 100,000 live births in developed countries [11]. Maternal mortality rate in Africa is the highest, and it is estimated at 500 deaths per 100, 000 live births. Fifty six percent (56%) occur in sub-Saharan Africa a region that accounts for only 21% of the world population [12]. In Ethiopia, maternal mortality still high. According to the World Bank report of 2018 the Ethiopian maternal mortality was 353 per 100, 0000 live birth [13].

Postpartum hemorrhage is one of the leading causes of maternal mortality and morbidity, accounted for approximately 27% of global maternal deaths, is defined as vaginal bleeding more than 500 ml after vaginal delivery, blood loss of more than 1000 ml after cesarean delivery and more than 1500 ml after cesarean hysterectomy [14].

In 2014 globally over 661,000(27.1%) maternal death was occurred due obstetric hemorrhage. More than two thirds of reported hemorrhage deaths were classified as postpartum hemorrhage. About 19.7% was occurred in developing countries. Eastern Asia accounted about 26.1% and in Sub-Saharan Africa around 200,000 maternal deaths was caused by postpartum hemorrhage [15].

According to the study conducted in Jimma University specialized hospital on the trend of maternal death from 2010 to 2014 most of maternal deaths occurred because of direct obstetric causes. Hemorrhage was the leading cause of death accounted about 54% in every year of the study, and it is the cause for more than half this death was occurred due to postpartum hemorrhage especially between 4 and 7 days of postpartum [16].

In addition to its direct cause of maternal of death, postpartum hemorrhage has also short and long term impacts like chronic illness, disability, increasing of death, poor growth and development of their children, hepatic dysfunction [17].

A woman suffering from hypovelimic shock secondary to postpartum hemorrhage can die within 2 h except she receives immediate blood transfusion and appropriate emergency obstetric care [18].

Non-pneumatic anti-shock garment is one of the first management tools for hypovolemic shock secondary to obstetric hemorrhage that is helpful in resuscitating and sustaining women in severe obstetric hemorrhagic shock. There was neither mortality nor clinically significant morbidity in this reappraisal so with out of this advice maternal mortality and morbidity very high [19].

The study conducted on the non-pneumatic anti-shock garment at Global Health study showed that with help of the non-pneumatic anti-shock garment for postpartum hemorrhage management blood loss decreased by 42–55%, vital signs and restoration of circulatory stabilized rapidly, maternal mortality was reduced by 66%, morbidity decreased by 44–66% and there were 32–73% lower extreme adverse outcomes (EAOs) [20]. At referral, tertiary-level facilities using non-pneumatic anti-shock garment for the management of postpartum hemorrhage reduce maternal mortality by 48% [21].

Study done in Ibadan Nigeria in 2014 utilization of non-pneumatic anti-shock garment for the management of post-partum hemorrhage was low, which was only 14.5% [22].

Low utilization of non-pneumatic anti-shock garment in Bayelsa State in which 46.4% of Midwives used for the management of postpartum hemorrhage this indicated low utilization of non-pneumatic anti-shock garment among respondents despite the efficacy of non-pneumatic anti-shock garment in reducing maternal morbidity and mortality related postpartum hemorrhage-related complication [23].

However, regardless of effective and many benefits associated with the use of Non-Pneumatic Anti shock garment in the management of postpartum hemorrhage its utilization is still very low. Even in Ethiopia still there is no any study showed utilization of non-pneumatic anti-shock garment for the management of postpartum hemorrhage by health care professionals. This study was therefore designed to assess health care professional utilization of non-pneumatic anti-shock garment and associated factors for the management of postpartum hemorrhage in Public hospitals of Jimma Zone South West Ethiopia.

## Methods and material

### Study area and the study period

The study was conducted in Jimma zone public hospitals, Oromia regional state south-West Ethiopia. Jimma zone far 350 Km from Addis Abbaba. The Jimma zone has a total area of 119,316 Square kilo meters. It has 21 woreda and 1 town administration with a total of 555 kebeles of which 515 of them are rural and 30 are urban. The population projection of 2014/15 of the zone is 3,090,112 with total reproductive age groups of 616,380.

In this zone there were seven public hospitals: Jimma medical center, Shanan Gibe hospital, Agaro Hospital, Limmu Hospital, Seka Hospital, Omo Nada Hospital and Satama Hospital. The study was conducted from April 01 to, 202,019.

### Study design

Facility based cross-sectional study design both quantitative and qualitative methods were employed.

### Source and study population

All health care professionals’ who were working in Jimma Zone public Hospitals were source of population. And all health care professionals who were working in Jimma Zone public Hospitals and fulfilled the selection criteria.

### Selection criterion

#### Sampling procedure

The study included a total of 210 included by census sampling technique in quantitative study. An in-depth interview was conducted on 10 key informants in qualitative. These health care professionals were chosen purposively based on their involvement in the use of NASG and Head of maternity ward. Participants who participated in in-depth interview were not involved in quantitative study.

#### Operational definitions and definitions of terms

##### Utilization of non-pneumatic anti-shock garment (NAG)

is measured based on the response to the question whether health care professionals used NASG for the management of postpartum hemorrhage at least one time [22, 24].

##### Knowledge scale

The respondents score of total knowledge questions those who below 50% were graded as having poor knowledge while those 50.0% and above were graded as having good knowledge [25].

Attitude scale: The attitude was considered “Positive” if the percentage score was 50% and above more and “Negative” if less than 50%.

### Measurements and data collection procedure

Aself-administered semi- structured questionnaire was used to collect data from study participants. The instrument was adapted and developed from different literatures [22–24, 26, 27]. It was prepered in English version and the questionnaire contains four parts which include socio demographic status eight questions, knowledge of Non-pneumatic anti-shock garment eight questions, attitude of health care professional regarding Non- pneumatic anti-shock garment eight questions and the responses consist five likert scales which were strongly agree, agree,neutral, disagree and strongly disagree and the fourth part was organizational (hospitals) related questions. The attitude statetment five likert scale for analysis was become three likert scale. Strongly agree and agree became agree and disagree and strongly disagree became disagree and neutral. Before the actual data collection, the questionnaire was pre-tested on 10% of health care professionals in Metu Karl Hospitals and appropriate corrections were made such as logical order of some questions, some words difficult to understand were revised. The overall standardized cronbach’s alpha for internal consistent or the reliability score of measurement was 0.86.

In-depth interview was carried out by the principal investigator and note taker on who agreed to be interviewed.

### Data analysis

Following the data collection, data were coded, and entered to a computer using Epidata version 3.1 and then exported to SPSS version 23.0 for analysis. Descriptive statistics and binary logistic regressions analysis were performed. In the binary logistic regression, both binary and multivariable analyses were carried out. All the variables were entered into bivariate analysis and those independent variables with a p- value < 0.25 in crude analysis was considered as a candidate for multivariable analysis and those variables with a *p* value < 0.05 in multivariable analysis were considered as statically significant of non-pneumatic anti-shock garment utilization. Multi-collinearity was checked to see the linear correlation among the independent variables by using the variance inflation factor and standard error. None of the variables yield inflation factor > 10 and standard error > 2 (VIF < 4.66 and Std. E < .137) and they were not dropped from multivariable analyses. Hosmer and Lemeshow’s test was found to be insignificant (*p*-value =0.158) and Omnibus test was significant (*p*-value = 0.000) which indicate that the model was fitted. Finally, the result of the analysis was presented in texts, tables and graphs as appropriate. For qualitative study, first for each individual tape recorded was transcribed and after reading again and again then data was translated. Then data were coded by number for each translated after data were coded similar numbers pooled together and the theme was developed. Finally presented in narratives & used to support the quantitative findings.

### Ethical considerations

Before data collection, ethical clearance was obtained from the institutional review board of Jimma University, Institute of health and submitted to each hospital. Permission was obtained from each hospital prior to data collection. The written consent was obtained from each participant that participation was voluntary and they have the right to withdraw at any time from the study. The informed consent was comprised the study purpose and procedures, potential risks and benefits, voluntary participation and right of withdrawal, the information provided by each respondent was kept strictly confidential.

## Results

### Socio demographic characteristics

A total of 220 questionnaires were distributed to the participants but only 210 were replied; the rest 10 did not fill the questionnaire correctly and excluded from the analysis with response rate of 95.5%.

The mean age of the respondents was 28.36 years. Half of participants 106 (50.5%) were in the age group of 25–29. Among the respondents, 121 (57.6%) were male. Nearly (51.9%) of respondents were married. Majority of them were Oromo by ethnicity (70.0%). Eighty six (41.0%) were Muslim by religion. Professionally 145 (69.0%) were Midwives. One hundred forty eight (70.5%) had 1–5 years of work experiences (Table 1).

**Table 1:** Socio-demographic characteristics of health care professional who were working in Jimma zone public Hospitals Southwest Ethiopia, April 2019

Variables	Frequency	Percentage
Age in years		
20–24	39	18.6
25–29	106	50.5
30–34	41	19.5
35–39	24	11.4
Sex		
Male	121	57.6
Female	89	42.4
Marital status		
Married	109	51.9
Single	101	48.1
Ethnicity		
Oromo	147	70.0
Amhara	33	15.7
Tigrai	14	6.7
Gurage	9	4.23
Others	7	3.33
Religion status		
Muslim	86	41.0
Orthodox	77	36.7
Protestant	41	19.5
Others	6	2.9
Profession		
Midwives	145	69.0
Nurses	46	21.9
Medical doctor	5	2.4
Emergency surgery	14	6.7
Educational level		
Diploma	50	23.7
Bachelor degree	142	67.6
Masters	14	6.7
Specialist	4	1.9
Years of experience		
1–5 years	148	70.5
6–10 years	55	26.2
11 years and above	7	3.3

Other: Ethnicity: Wolayita, Hadiya, Silte, Kafa

Religion: Wakefata, Catholic

### Respondents’ knowledge of non-pneumatic anti-shock garment

This study indicated that, majority (80.0%) of respondents had heard about non-pneumatic anti-shock garment while 42 (20.0%) never heard before. Ninety one of the respondents heard from hospital (54.2. %). Ninety eight (58.3%) of the respondents said that non-pneumatic anti-shock looks like trousers and 55 (32.7%) said bottom half of suit. Ninety seven (57.7%) of the respondents correctly mentioned that non-pneumatic anti-shock garment has six parts. Regarding the function of Non-pneumatic anti-shock garment, 21.0% mentioned that non-pneumatic anti-shock garment increases blood flow to vital organs. Concerning the criteria for the application of non-pneumatic anti-shock garment for women with postpartum hemorrhage about 98 (36.8%) said NASG applied when blood loss was greater than 750 ml, (Table 2).

**Table 2:** Knowledge of NASG among health care professionals who were working in Jimma zone public Hospitals Southwest Ethiopia, April 2019

Variables	Frequency	Percentage
Known NASG as it used for pph complication mgt		
Yes	168	80.0
No	42	20.0
Source of information (first)		
Training	36	21.4
Conference	6	3.6
Hospital	91	54.2
From textbook	26	15.5
Electronics	4	2.4
Other	5	3.0
NASG look like		
Gown	15	8.9
Trousers	98	58.3
Bottom half of suit	55	32.7
How many parts have the NASG		
Four	39	23.2
Six	97	57.7
Eight	24	14.3
Nine	8	4.8
What is the function NASG? *		
Prevent Shock	97	16.7
Stabilize the women in shock	104	17.9
Reverse shock	54	9.3
Decrease blood loss	98	16.8
Compress blood vessels	91	15.6
Increase blood flow to vital organs	122	21.0
No response	8	1.4
Other	7	1.2
Know proper use of NASG		
Yes	122	72.6
No	46	27.4
Total	168	100.0
Non-pneumatic anti-shock garment applied for women with PPH when*		
Bleeding > 750 ml	98	36.8
systolic blood pressure < 90 mm	84	31.6
Pulse > 110 bpm	84	31.6
NASG removed when		
Blood loss < 50 ml/hr.,	71	21.8
Pulse < 100 bpm	88	27.1
Systolic blood pressure > 100 mmhg	90	27.7
Normal shock index	76	23.4
Good Knowledge	100	59. 5
Poor knowledge	68	40.5

^*^Multiple responses are possible

About 59.5% respondents’ had good knowledge towards non-pneumatic anti-shock garment.

### Attitudes of health care professionals towards utilization of non-pneumatic anti shock garment

Regarding to the attitudes of the respondents towards utilization of NASG for the management of postpartum hemorrhage, about one hundred forty seven (70%) of the respondents agreed that NASG is necessary for the management of postpartum hemorrhage in all settings and 5.7% disagree. One hundred forty two (67.6%) respondents agreed that NASG can used along with standard treatment protocols of postpartum hemorrhage management and 9 (4.3%) disagreed. Concerning the time required to apply NASG (Table 3).

**Table 3:** Attitudes towards NASG utilization among health care professionals who were working in Jimma zone public Hospitals Southwest Ethiopia, April 2019

Variables	Agree	neutral	Disagree
	No (%)	No (%)	No (%)
The use of non-pneumatic anti shock garment is necessary for the management of postpartum hemorrhage in all settings.	147 (70)	49 (23.3)	14 (6.7)
NASG used along with standard treatment protocols of postpartum hemorrhage.	143 (68.1)	55 (26.2)	12 (5.7)
NASG can be applied with minimum procedures in short period of time.	100 (47.6)	55 (26.2)	55 (26.2)
Removal NASG requires a lot of procedures that takes time.	120 (57.1)	40 (19.1)	50 (23.8)
Anti-shock garment is only beneficial to people in the rural areas/primary care settings	28 (13.3)	55 (26.2)	127 (60.5)
Manual removal of placenta is possible with NASG in place	141 (67.1)	43 (20.5)	26 (12.4)
Anti-shock garment is effective in patients with cervical lacerations	104 (49.5)	67 (31.9)	39 (18.6)
The garment should be a must in every health care facility that has maternity service	144 (68.6)	35 (16.7)	31 (14.8)

Regarding the attitudes of respondents towards non-pneumatic anti-shock garment utilization more than half of (53.58%) had positive attitudes.

### Distribution of health care professionals by their organization (hospitals)

Regarding the organizational factors, 87 (41.4%%) respondents were working at general hospitals, 126 (60%) of the respondents said that their hospitals have two and above number of non-pneumatic anti-shock garment. About 59 (28.1%) respondents have training on non-pneumatic anti-shock garment utilization (Table 4).

**Table 4:** Distribution of health care professionals by their organizations who were working in Jimma zone public Hospitals Southwest Ethiopia, April 2019

Variables	Frequency	Percentage
What is the level of your hospital?		
Primary hospital	58	27.6
General hospital	87	41.4
Tertiary hospital	65	31
Received training on NASG		
Yes	59	28.1
No	151	71.9
How many numbers NASG available at your hospital?		
One	84	40
Two and above	126	60
Where does NASG placed after use in your hospital?		
In labor ward	124	59
In not labor ward	86	41

### Utilization of non-pneumatic anti-shock garment

Concerning the utilization of non-pneumatic anti-shock garment by health professionals, seventy six (36.2%) used NASG for postpartum hemorrhage management while 63.8% never applied it for postpartum hemorrhage complication management. The reasons stated by respondents those did not use non-pneumatic anti-shock garment, (35.4%) were due to lack of experience and (23.8%) due to lack of awareness about availability of the instruments (Table 5).

**Table 5:** Utilization of non- pneumatic anti-shock garment among health care professionals who were working in public Hospitals of Jimma Zone South West Ethiopia, April 2019

Variables	Frequency	Percentage
Used NASG for the management of PPH?		
Yes	59	36.2
No	134	63.8
Do you use NASG every time there is PPH?		
Yes	32	15.24
No	43	20.48
Never use	134	63.81
If no, when do you use it?*		
Severe PPH	41	38.3
Shock	43	40.2
When other method is fail	23	21.5
Do you use it, when the need arise in your hospital?		
Yes	62	29.52
No	14	6.67
Never use	134	63.81
If no, why? *		
It is difficult to assemble	10	41.7
It is not ready available for using	9	37.5
I do not know much about it	5	20.8
Reason for non-utilization*		
Availability of other method	30	14.3
Effective management of third stage of labor	28	21.4
Lack of experience	58	27.6
Not aware existence of NASG in the hospital	39	18.6
The garment is not convenient	9	4.3

*Multiple responses are possible

### Factors associated with utilization of non-pneumatic anti-shock garment

The bivariate analysis result revealed that types of professions, educational status, knowledge of respondents, attitude of respondents, training on NASG, types of hospitals, number of NASG and place where placed after use were significantly associated utilization of NASG.

All the significant independent variables in a bivariate analysis were entered together in a multivariable logistic regression using backward method to determine final predictors of utilization of non-pneumatic anti-shock garment controlling for potential confounders. Accordingly; knowledge level, attitude, having training on NASG, number of NASGs hospitals have were independent predictors of utilization of non-pneumatic anti-shock garments. In this study health care professionals who had a good knowledge about NASG were 3.957 times more likely to use the NASG as compared to health professionals who had poor knowledge about NASG[AOR = 3.957, 95% CI:(1.67, 9.407, *p* = 0.002)].

This finding was supported by qualitative finding*.*

A health care provider working at labor ward explained NASG as; *I have used NASG for the management of postpartum hemorrhage* many times. NASG is not a drug but it is cloth used for postpartum hemorrhage management. It stabilizes woman in shock, increase blood flow to vital organ and decrease blood loss. It is not simple removed after applied. To remove it there is a rule called rule of 20 after measuring the vital sign blood pressure and pulse in normal range at least for 2 hours then each segment should be removed every 15 min starting from segment one to six. If the vital sign changes by **20** in between removing means pulse increased by 20 bpm and BP decreased by 20 mmhg, the NASG should be reapplied.

Regarding the attitudes of health care professionals those who had positive attitude toward the NASG utilization were 3.54 times more likely to use NASG than those who had negative attitude towards NASG utilization for postpartum hemorrhage management [(AOR = 3.54, 95% CI:(1. 37, 9.13, *p* = 0.009)].

…. Staff working at labor said that “I have the knowledge of the non-pneumatic anti-shock garment as it used for postpartum hemorrhage to reverse shock and stabilize the women condition but I didn’t use it because I preferred other methods like normal saline, warm cloth and blood transfusion for woman with postpartum haemrrahge.”

Concerned with status of training by health care professionals on NASG use, those health professionals who received training on the use of NASG were 13.156 times more likely use NASG as compared with who didn’t received training on NASG use (AOR: 13.156, 95% CI:(4.89, 36.00, *p* = 0.0001)]. This is supported by qualitative finding. For instance:

… A 25 years old health professional said that “I didn’t use NASG for the management of postpartum hemorrhage complication before because I have no training and experience how to use it. If I receive training I use it without any difficult because I have seen the health professionals who were trained can apply and remove easily.”

Relating to NASG, those health care professional whose hospitals had two and above NASGs, were 8.7 times more likely to use NASGs as compared with those health professionals’ hospital had only one NASG for postpartum hemorrhage management of complication (AOR: 8.70, 95% CI: (2.89, 26.20, *p* = 0.0001)] (Table 6).

**Table 6:** Multivariable logistic regression showing independent factors of utilization of non-pneumatic anti-shock garment among health care professionals working in Jimma zone public Hospitals South West Ethiopia, April, 2019

Variables	Categories	Utilized NASG		COR (95%CI)	AOR (95%CI)	p- value
		Yes N (%)	No N (%)			
General Knowledge	Good knowledge	61 (36.31%)	39 (23.21%)	5.53 (2.74, 11.13) 1.0	3.967 (1.67, 9.407) 1.0	0.002
	Poor knowledge	15 (8.93%)	53 (31.55%)			
Total Attitude	positive attitude	66 (31.4%)	47 (22.4%)	12.212 (5.75, 25.96)	3.540 (1.37, 9.13) 1.0	0.009
	Negative attitude	10 (4.8%)	87 (41.4%)	1.0		
Training status	Yes	47 (22.4%)	12 (5.7%)	16.477 (7.77, 34.96)	13.156 (4.81, 36.00)	0.0001
	No	29 (19.21%)	122 (80.79%)	1.0	1.0	
NASG number	One	13 (6.2%)	71 (33.8%)	1.0	1.0	0.0001
	Two and above	63 (30%)	63 (30%)	5.461 (2.75, 10.85)	8.70 (2.89, 26.20)	

This finding was supported by qualitative finding. For instance:

…. A 32 year head of maternity ward said that, previous we have three non-pneumatic anti-shock garments that our staffs applied for the mothers who have postpartum hemorrhage. But now there is only one NASG at our hospital and the others were referred mother to referral hospital by NASG that they never gave back for us and left there. Currently we rarely used NAG because when NASG we have applied for the mother and other presented with similar case we never applied it because we have only one and also before the NASG is cleaned we never use it that mother presented without NASG.

## Discussion

Non-pneumatic anti-shock garment (NASG) can prevent the postpartum hemorrhage complication by decreasing blood flow to the lower extremities and increasing blood flow to vital organs like brain, heart, and lung.

Based on this finding 36.2% of the respondents utilized the non-pneumatic anti-shock garment on the client for the management of postpartum hemorrhage. This finding was almost similar finding in Ibadan Nigeria and Sokoto State Specialist Hospital (35%) in both study areas [24, 26]. How every it was higher when compared with study done in Ondo state Nigeria that 14.1% of respondents’ utilized non-pneumatic anti-shock garment for postpartum hemorrhage management [22]. The difference may be due sample size difference, sampling technique used. On the contrary it was lower compared to finding in Bayelsa State Nigeria and Benin Central Hospital that 46.4 and 42% of the respondents used non-pneumatic anti-shock garment for postpartum hemorrhage management respectively [23, 25]. The slight difference in utilization might be due to difference between study participants in which only Midwives participated in case of study conducted in both study areas and also may be due to variation in sampling technique used.

This study also revealed that knowledge, attitude, non-pneumatic anti-shock garment number, training status of the respondents and their utilization of NASG had significant association with *P*-value < 0.05.

Good utilization of non-pneumatic anti-shock garment was seen among health care professionals who had good knowledge about NASG. Those who have good knowledge utilize non-pneumatic anti-shock garment about four times more likely as compared to having poor knowledge. This implies that good knowledge exerts a positive effect on the extent of health care professionals’ utilization of NASG. This is supported by the study conducted in Ondo State and Benin Central Hospital, that there was association between respondents’ knowledge and their utilization of non-pneumatic anti-shock garment [25, 27]. This may be due to the fact that good knowledge improves the confidence and readiness of the health care professionals’ to utilize NASG for postpartum hemorrhage management and increase their ability to use it appropriately. However it was differ from Bayelsa State Nigeria that there was no significant association between respondents’ knowledge and their utilization of non-pneumatic anti-shock garment [23]. The discrepancy may be due the difference of sample size, this study included different types of health care professionals.

Another independent factor showed that there was more utilization of NASG for postpartum haemorrhage management among health care professionals who had positive attitude. This implies that a positive attitude regarding NASG exerts a positive effect on the extent of health care professionals’ NASG utilization practice. Health care professionals’ have positive attitude there is a big tendency to utilize properly. It is different from the study finding in Bayelsa State Nigeria that there is no association between respondents’ attitude and non-pneumatic anti-shock garment utilization [27]. This difference may be due to difference in variable categorization, variation in setting from place to place, may be due sampling technique used for sample selection (SRS) and may also due variation in the population participated in the study.

This study showed that good utilization of non-pneumatic anti-shock garment was seen among health care professionals who took training on non-pneumatic anti-shock garment as compared to those who have no training. Those health professionals who trained on non-pneumatic anti-shock were about thirteen times more likely used than those who had no training. This may be due to the fact that attending training can help health care professionals to get more knowledge on how to apply, remove non-pneumatic anti-shock garment and this can improve their utilization of non-pneumatic anti-shock garment.

In this study non-pneumatic anti-shock garment number was other factor that statically associated with utilization of non-pneumatic anti shock garment by health care professionals. Hospitals having two and above non-pneumatic anti-shock garment were around nine times more likely used than those who have one.

Health care professionals play a vital role in the reduction of maternal mortality and morbidity in all health facilities. Postpartum hemorrhage is one of the leading causes of maternal mortality especially in the developing countries. It is therefore, important that health care providers are always abreast with current techniques and equipment used in preventing and management of postpartum hemorrhage. This study showed low utilization of non-pneumatic anti shock garment among the health care providers, and this important concept included in maternal health program and policy planners.

### Strength and limitations

The study involved both quantitative and qualitative methods of data collections to maximize the reliability of the data collected. The questionnaire was prone to social desirability bias; hence it assesses self-reported regarding utilization of NASG, there might be over-reporting of a behavior. Recall bias was there because the majority of the health care professionals were asked about their past utilization of NASG. In addition to these, the lack study in Ethiopia and adequate studies did out of Ethiopia on this topic make comparison and discussion difficult.

### Conclusion and recommendation

This study showed that of the respondents’ utilized non-pneumatic anti shock garment for the management of post-partum hemorrhage low. The study also identified factors associated utilization of non-pneumatic anti-shock garment. The independent factors associated with utilization of non-pneumatic anti-shock garment were having good knowledge of health professional; attend training on non-pneumatic anti-shock garment, positive attitudes towards non-pneumatic anti-shock garment utilization and more number of non-pneumatic anti-shock garments available. Periodic training for health professionals on the use of non-pneumonic anti-shock garment for management of obstetric hemorrhage is useful.

## Data Availability

The spreadsheet data supporting the findings of this work is available at the hands of the corresponding authors.

## Abbreviations

ATSM: Active third stage management
EAO: Extreme adverse outcome
FIGO: Federation of Gynecology and Obstetrics
ICM: International Confederation of Midwives
NASG: Non-pneumatic Anti-shock Garment
PPH: Postpartum hemorrhage
